# Using data-driven approaches to improve delivery of animal health care interventions for public health

**DOI:** 10.1073/pnas.2003722118

**Published:** 2021-01-18

**Authors:** Stella Mazeri, Jordana L. Burdon Bailey, Dagmar Mayer, Patrick Chikungwa, Julius Chulu, Paul Orion Grossman, Frederic Lohr, Andrew D. Gibson, Ian G. Handel, Barend M. deC. Bronsvoort, Luke Gamble, Richard J. Mellanby

**Affiliations:** ^a^The Epidemiology, Economics and Risk Assessment Group, The Roslin Institute and The Royal (Dick) School of Veterinary Studies, The University of Edinburgh, Easter Bush Veterinary Centre, Roslin EH25 9RG, United Kingdom;; ^b^Mission Rabies, Blantyre, Malawi;; ^c^Worldwide Veterinary Service, Blantyre, Malawi;; ^d^Department of Animal Health and Livestock Development, Lilongwe, Malawi;; ^e^Mission Rabies, Cranborne BH21 5PZ, United Kingdom;; ^f^Division of Veterinary Clinical Studies, The Royal (Dick) School of Veterinary Studies, The University of Edinburgh, Hospital for Small Animals, Easter Bush Veterinary Centre, Roslin EH25 9RG, United Kingdom

**Keywords:** rabies, data-driven, Malawi, zoonosis, vaccination

## Abstract

Rabies kills ∼60,000 people per year. Annual vaccination of at least 70% of dogs has been shown to eliminate rabies in both human and canine populations. However, delivery of large-scale mass dog vaccination campaigns remains a challenge in many rabies-endemic countries. In sub-Saharan Africa, where the vast majority of dogs are owned, mass vaccination campaigns have typically depended on a combination of static point (SP) and door-to-door (D2D) approaches since SP-only campaigns often fail to achieve 70% vaccination coverage. However, D2D approaches are expensive, labor-intensive, and logistically challenging, raising the need to develop approaches that increase attendance at SPs. Here, we report a real-time, data-driven approach to improve efficiency of an urban dog vaccination campaign. Historically, we vaccinated ∼35,000 dogs in Blantyre city, Malawi, every year over a 20-d period each year using combined fixed SP (FSP) and D2D approaches. To enhance cost effectiveness, we used our historical vaccination dataset to define the barriers to FSP attendance. Guided by these insights, we redesigned our vaccination campaign by increasing the number of FSPs and eliminating the expensive and labor-intensive D2D component. Combined with roaming SPs, whose locations were defined through the real-time analysis of vaccination coverage data, this approach resulted in the vaccination of near-identical numbers of dogs in only 11 d. This approach has the potential to act as a template for successful and sustainable future urban SP-only dog vaccination campaigns.

## Significance

Rabies is arguably the exemplar of the One Health Agenda in which preventative health care in one species can improve health of other species. Interrogation of large epidemiology datasets offers the potential to deliver health care initiatives in a more efficient and cost-effective manner. However, real-life examples demonstrating this potential are limited. Here, we report a real-time, data-driven approach to improve cost effectiveness of dog vaccination campaigns in urban sub-Saharan African settings, which eliminates the need of expensive door-to-door vaccination by replacing them with strategically positioned fixed and roaming static points (SPs). This approach has the potential to act as a template for future successful and sustainable urban SP-only dog vaccination campaigns.

Rabies is a devastating disease that causes around 59,000 human deaths annually, creating an economic burden estimated at $8.6 billion per annum (1). The World Health Organization, World Organization for Animal Health, and Food and Agriculture Organization have set the goal of eliminating dog-mediated human rabies deaths by 2030. This could be achieved through mass administration of postexposure prophylaxis (PEP) to human dog bite victims, elimination of dog rabies by mass dog vaccination, or a combination of both approaches (2). Importantly, mass dog vaccination increases the cost efficiency of rabies elimination campaigns compared to the use of PEP alone (3–5). While regions such as Central and South America have made successful steps toward rabies control, with several countries declared free of human cases of dog-transmitted rabies (6), sub-Saharan Africa (SSA) and India still carry a disproportionately large part of the global burden of rabies (1).

As almost all human rabies cases are caused by bites from rabies-infected dogs (7), rabies is preventable through mass dog vaccination. Annual vaccination of at least 70% of the dog population has been shown to be highly effective at reducing rabies incidence in both human and dog populations and is recommended by the World Health Organization for countries working toward rabies elimination (8, 9). Despite this recommendation being in place for a number of years, global rabies elimination programs have floundered due to a diverse mix of political, technical, and logistical challenges.

According to the 2018 census (10), Malawi has a population close to 18 million people and a dog population close to 1.5 million based on a pan-African human:dog ratio of 21.20:1 (11). Rabies is a major public health issue in Malawi, where there are estimated to be 500 human rabies deaths and economic losses of 13 million USD per year (1). The Animal Health and Livestock Development Policy in Malawi (12) recognizes rabies as an endemic disease and recommends that at least 80% of dogs should be vaccinated every year to control rabies, and that vaccination should be provided for free during rabies campaign periods (13). Nevertheless, the countrywide dog rabies vaccination coverage has been estimated to be very low (1), with only small pockets of high vaccination achieved through localized nongovernmental organization (NGO) campaigns.

To date, the most successful vaccination programs, which have achieved vaccination coverages greater than 70% in Malawi (14) and other SSA countries, have been dependent on a combined static point (SP) and door-to-door (D2D) approach (14–18). Fixed SP (FSP) methodology, whereby a predefined location is advertised and dog owners are requested to bring their dogs for vaccination, can be highly cost-effective in some low- and middle-income countries where the majority of dogs are owned and can be handled, restrained, and brought to FSPs. However, it often fails to achieve adequately high vaccination coverages. For this reason, one-off FSP vaccination approaches often need to be supplemented with additional approaches, which include costly, time-consuming, and logistically challenging D2D visits (14, 15) or people repeating FSP vaccinations in areas where adequate vaccination coverage had not been reached following one-off FSP approaches (16, 17). The combination of low attendance at FSPs and subsequent requirement for dog vaccination teams to visit all households in D2D campaigns hinders the scalability of dog vaccination campaigns from localized regional campaigns to national elimination programs. Consequently, there is a clear need to develop campaigns which have a high attendance at FSPs, thereby eliminating the need to have subsequent D2D campaigns.

Since 2015, the NGO Mission Rabies has been conducting annual mass dog vaccination campaigns in Blantyre city, Malawi, using a combination of FSPs and D2D campaigns, which have been consistently successful in vaccinating large numbers of dogs at over 70% vaccination coverage based on postvaccination surveys (PVSs) covering a sample of the whole city. Using data gathered during these campaigns, we have established the reasons why dog owners do not attend FSPs in Blantyre city. We have found that distance from household to FSP plays a decisive role in the decision of a dog owner to attend an FSP, with very few people willing to travel more than 1.5 km to bring their dog for vaccination. Furthermore, our multivariable logistic regression model indicated that dogs from poorer areas had a greater chance to be brought to an FSP for vaccination, while puppies and pregnant/lactating dogs were less likely to be brought to an FSP. We also investigated why owners did not attend FSPs through a questionnaire delivered to over 11,000 owners. This demonstrated that, in addition to factors identified by the model, owners reported lack of awareness about the campaign and difficulty in handling dogs as important reasons for not attending an FSP (18).

These findings allowed us to take a data-driven, evidence-based approach to redesign our 2018 Blantyre city vaccination campaign using SPs only. This paper describes our innovative, data-driven approach, which allowed us to achieve an adequate vaccination coverage in a large city-wide campaign in SSA without the need for a costly, labor-intensive, and logistically challenging D2D component. Our redeveloped dog vaccination campaign was able to achieve above 70% vaccination coverage among a population of over 40,000 dogs in 11 d compared to 20 d using only FSPs and roaming SPs (RSPs). Our work is a powerful example of how big epidemiology datasets can be interrogated to provide insights that drive improvements in the cost-effectiveness of public health campaigns. This work provides a template of scalable mass dog vaccination strategies in SSA.

## Results

### Vaccination Campaigns.

The total numbers of dogs vaccinated during the 2015, 2016, and 2017 20-d Blantyre city campaigns were 35,216, 34,783, and 34,132, respectively. Totals of 23,442, 24,538, and 24,614 dogs were vaccinated at FSPs, and 11,774, 10,245, and 9,521 were vaccinated D2D, in 2015, 2016, and 2017, respectively.

In comparison, during the 11-d 2018 Blantyre city campaign using the modified approach of FSPs and RSPs, a total of 33,000 dogs were vaccinated, 30,074 at FSPs and 2,926 at RSPs. In other words, attendance to FSPs increased by 22% between 2017 and 2018 against the background of very similar FSP attendance levels during the previous 3 y.

The success of the revised approach taken in 2018 was repeated in 2019 and 2020, when the dual approach of FSPs and RSPs achieved vaccination coverages of 75% and 76%, respectively. In 2019, a total of 32,317 dogs were vaccinated across a combination of FSPs and RSPs, and, in 2020, 37,815 dogs were vaccinated using a similar combination of FSPs and RSPs.

### Interim PVSs.

The overall coverage achieved after 8 d of FSPs was estimated as 65% based on interim PVSs in 110 working zones. Twenty-six of 110 working zones surveyed (24%) had estimated coverage of less than 50%. This classification was used as an outcome variable in the regression model described below to identify which zones among those not surveyed were likely to have low vaccination coverage and therefore should be revisited with RSPs.

### Predicting Areas of Low Coverage.

**Fig. 1.**
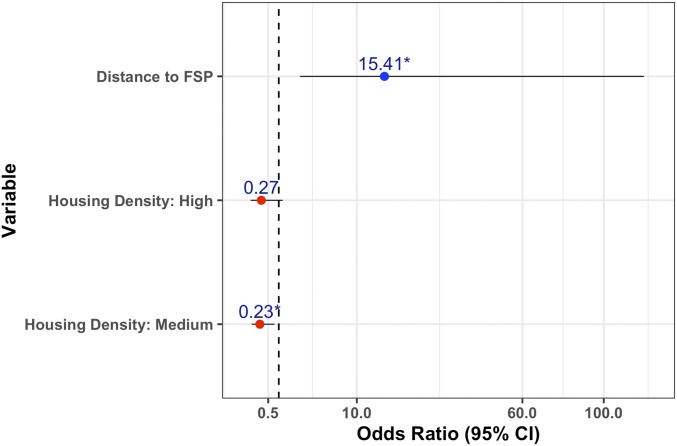
Regression model predicting zones with low vaccination coverage. Results of the multivariable logistic regression model predicting areas of poor vaccination coverage, including odds ratios and 95% confidence intervals for each variable.

The multivariable logistic regression model selection procedure is shown in Table 1. Model one was chosen as the final regression model due to having the lowest Akaike information criterion (AIC), high predictive ability (ranked fifth), and simplicity. Moran’s *I* test at binned distances between vaccination zones indicated absence of residual spatial autocorrelation, as all *P* values were greater than 0.05 (*SI Appendix*, Fig. S5). The regression model (Fig. 1 and *SI Appendix*, Table S4) showed that areas of low housing density and areas furthest away from the nearest FSP were more likely to have poor vaccination coverage. In addition to any areas identified during the PVSs as having a coverage of less than 60% (*n* = 37), 22 zones were chosen by the model to be revisited with RSPs during the fifth weekend of the campaign (vaccination days 9 to 11).

**Table 1.** Model selection procedure

Index	Model	AIC	AUC
1	housedens + dist2sp	95	0.83
2	dist2sp	97	0.79
3	housedens + dist2sp + housedens × dist2sp	98	0.82
4	pop4 + dist2sp	98	0.83
5	housedens + pov4 + dist2sp	99	0.85
6	housedens + pop4 + dist2sp	100	0.82
7	housedens + dist2sp + dist2sp × pop4	100	0.82
8	housedens + dist2sp + dist2sp × pov4	100	0.83
9	pov4 + pop4 + dist2sp	102	0.84
10	housedens	102	0.75
11	housedens + pov4 + pop4 + dist2sp	104	0.85
12	pop4	105	0.76
13	housedens + pop4	106	0.77
14	housedens + pov4	106	0.78
15	pov4 + pop4	109	0.77
16	housedens + pov4 + pop4	110	0.78
17	pov4	120	0.64

Candidate models are arranged by AIC and AUC. dist2sp, distance to FSP; housedens, housing density; pop4, population density; pov4, poverty.

### Final PVSs.

**Fig. 2.**
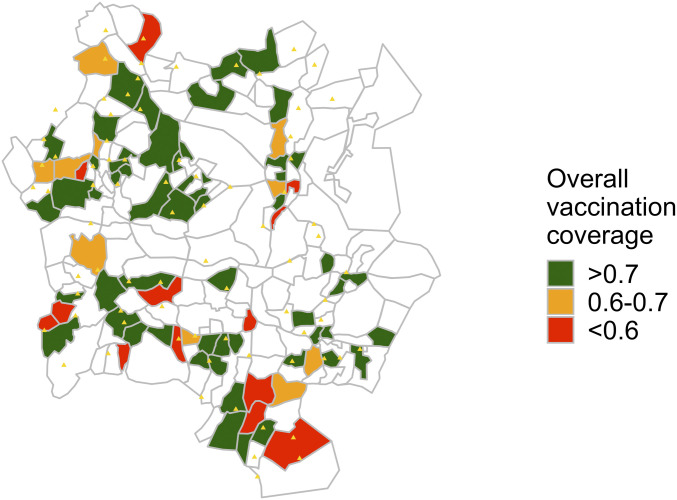
Final vaccination coverage estimates. Vaccination coverage estimates for each randomly selected zone surveyed during the final PVSs.

At the end of the 11-d campaign, PVSs were repeated to assess the overall success of the campaign. A total of 79 working zones were surveyed. Results of the overall vaccination coverage assessment estimated an overall vaccination coverage of 79%, which ranged from 39 to 100% per zone (Fig. 2), with only 4 of the 79 (5%) zones having a coverage less than 50%.

### Comparison of Vaccination Campaign Logistics.

The numbers of dogs vaccinated per campaign day and per person per campaign day are shown in Table 2. During the 2017 campaign, on average, 3,077 dogs were vaccinated at FSPs and 793 dogs during D2D per day. In comparison, during the 2018 campaign, the average number of dogs vaccinated at FSPs increased to 3,759 dogs per day, and 975 dogs were vaccinated during RSPs per day.

**Table 2.** Comparison of number of dogs vaccinated between the 2017 and 2018 campaigns

Year	Type	No. of dogs vaccinated	Dogs vaccinated per person-day	Dogs vaccinated per day
2017	FSP	24,614	46.62	3,077
2017	D2D	9,521	12.02	793
2018	FSP	30,074	96.39	3,759
2018	RSP	2,926	49.59	975

Table 3 shows the number of personnel typically involved in the 2015 to 2017 campaigns compared to the personnel involved in the 2018 campaign. Crucially, the 2018 campaign required 904 person-days to deliver the program, whereas the 2015 to 2017 campaigns required 1,719 person-days. In addition, this was against only a modest rise in the number of people involved in sensitization and education programs, which increased from 320 person-days in previous campaigns to 389 in 2018. Since staff costs are an important variable cost of canine vaccination campaigns, these data unambiguously demonstrate the dramatic reduction in staffing required to deliver a successful vaccination campaign, both in terms of vaccination numbers and vaccination coverage following the implementation of our data-driven approach.

**Table 3.** Number of people involved in the 2018 vaccination campaign vs. previous years

Year	Activity	Details	No. of small teams	No. of people	No. of days	No. of person-days
2018	FSP	Weekend 1 and 2	14	42	4	168
	FSP	Weekend 3 and 4	12	36	4	144
	RSP	Weekend 5	10.5	21	2	42
	RSP	Monday	10	17	1	17
	Sens	Sensitization	—	7	11	77
	Sens	Sensitization	—	8	1	8
	Sens	By education team	—	16	1	16
	Educ	Education teams informed students about campaign	—	16	18	288
	PVS	Postvaccination surveys	—	10	3	30
	PVS	Postvaccination surveys	—	12	3	36
	PVS	Postvaccination surveys	—	13	6	78
Total						904
2015–2017	FSP	Weeks 1 and 2	22	66	4	264
	D2D	Weeks 1 and 2	22	66	6	396
	FSP	Weeks 3 and 4	22	66	4	264
	D2D	Weeks 3 and 4	22	66	6	396
	Educ	Education teams informed students about campaign	—	16	20	320
	PVS	Postvaccination surveys	—	9	2	18
	PVS	Postvaccination surveys	—	10	5	50
	PVS	Postvaccination surveys	—	11	1	11
Total	—	—	—	—	—	1,719

Educ, education; Sens, sensitization.

## Discussion

Our study highlights the persistent challenges of vaccinating high numbers of dogs at high coverage over a short time span using SPs alone. Our study site was a well-established field site where we had vaccinated over 70% of dogs annually in the previous 3 y using a combined FSP and D2D approach, with the campaign gaining increasing local engagement and support. Despite using an evidenced-based approach and modifying our FSP number and position, guided by analysis of large operational datasets collected in previous years, we still could not access over 70% of the dog population with FSPs alone. This revised approach involved a near doubling of FSPs and ensuring that nearly 80% of dog owners had access to an FSP within 812 m, the average distance people were willing to travel to an FSP based on our previous investigations of barriers to FSP attendance in the same city. To prevent a return to the D2D approach, we adopted an innovative approach, which maximized the workforce effectively to ensure that over 70% of dogs were vaccinated in this locality in a very short time frame. Our study is a striking example of how field epidemiological data can be interrogated in real time to guide swift and cost-effective field health care interventions. Although focused on rabies, this approach may be informative for other disease scenarios.

Rabies is a globally important and highly neglected tropical disease that continues to impose a devastating health and financial burden on some of the world’s poorest countries (1). Rabies is arguably the exemplar of the One Health Agenda in which preventative health care in one species can improve health of other species, and the absolute cost of eliminating this disease is less than the 1-y global cost of the disease. Furthermore, a combined dog vaccination/PEP approach has been shown to be more cost efficient than PEP alone. This is because PEP alone will lead to ever-increasing cumulative cost, while effective mass dog vaccination can interrupt transmission to both dogs and humans, therefore reducing the need for PEP (5). In the Blantyre region, 10 cases of pediatric rabies deaths were reported over a 9-mo period in 2011 and 2012 (19). Over the following 3 y prior to the start of our long-term vaccination program (May 2012 to May 2015), 12 children died from rabies. Since the start of our campaign in May 2015, a total of 2 children have died from rabies in the same geographical area around Blantyre over the subsequent 2 y (May 2015 to May 2017) (20), providing compelling evidence that our collaborative program of work is decreasing the incidence of pediatric rabies in the Blantyre region. It has been estimated that, if dog vaccination and PEP provision levels do not increase, more than 1 million people will lose their lives to rabies between 2020 and 2035 (21). Moreover, the status quo of PEP supply, whereby treatment costs are the responsibility of the bite victims and their families, who are often unable to pay in the face of this life-threatening disease, has been characterized as unjust, unfair, and unworkable (22). This health care failure highlights the need for urgent action in this space, particularly as a highly effective preventive vaccine has been available for more than a century (23).

Mass dog vaccination remains the most cost-effective strategy to eliminate canine-mediated human rabies in low- and middle-income countries (5, 24), yet coordinated, country-wide vaccination campaigns have been scarce in SSA. One of the reasons why rabies remains a major cause of mortality, despite having all the tools available to eliminate it, is because of the challenges of vaccinating dogs at high coverages at a national scale (25). These challenges include financial constraints, lack of expertise in campaign design, erroneous exclusion of puppies, limited surveillance, and lack of cooperation between the veterinary and health sector (26). Improving the scalability of dog vaccination campaigns has been the focus of our group’s activity over the past decade, and we have recently described high-number, high-coverage vaccination campaigns in numerous countries, including India, Uganda, Sri Lanka, and Malawi (14, 27–29).

The increase in ease of collection of field health care data through technology innovations such as mobile apps has resulted in increased availability of large health-related datasets. To date, these have been greatly underutilized due to lack of time and/or expertise (30). Our NGO/academic partnership aims to harness the availability of such datasets to optimize the rollout and evaluation of novel health care initiatives (31). In SSA, the vast majority of dogs are owned (14, 18, 32). This lends itself to mass dog vaccination through SPs, the most cost-efficient vaccination delivery method. However, to date, most campaigns in SSA have failed to deliver high-coverage vaccination without the D2D method, which is expensive, time-consuming, and labor-intensive. We have therefore taken a data-driven mixed-methods approach to understand the barriers to FSP attendance in our previous work (18) and have conducted a large-scale trial to assess the effect of optimizing the number and location of FSPs, with the aim of improving efficiency of mass dog rabies vaccination campaigns in SSA urban settings. This has resulted in a highly cost-effective health care intervention, both in terms of a significant reduction in number of staff and a vast reduction in number of total days required, with no compromise to absolute numbers of dogs vaccinated or vaccination coverage.

A key strength of our approach is the use of mobile phone technology to gather data, which supports an evidence-based approach to improve the delivery of health care interventions. The use of mobile phone app technology is rapidly growing in SSA. The app that we developed has now recorded over 1 million dog vaccinations in 10 countries around the world (33), and enabled us to map in detail where the FSPs and health care interventions were and then understand the impact of a range of geospatial and socioeconomical variables. This has then allowed us to roll out this revised, evidence-guided approach, which has resulted in significant advances in health care delivery. We showed that, by increasing the number of FSPs from 44 to 77 and optimizing their locations so that at least 77% of the dogs were within 812 m of the FSP—with 812 m being the mean straight-line distance people are willing to walk to reach an FSP as estimated by our previous work (18)—we have been able to achieve an increase in FSP attendance and a 65% dog vaccination coverage. We then used data collected through our interim PVSs to identify areas that would benefit from RSPs. This combination of FSPs and targeted RSPs resulted in the vaccination of 79% of the dog population in almost half the time compared to previous years and with a reduction in staff numbers. It is important to highlight that the distance dog owners are prepared to travel to attend an SP varies between countries, and this country-specific threshold should be borne in mind when planning vaccination campaigns (34, 35).

To achieve large-scale coordinated vaccination efforts in SSA that can be sustainably funded, there is an urgent need to find alternative approaches to expensive D2D vaccination methods. Barriers to successful FSP campaigns have been investigated by a number of studies (15, 34, 36–40). This study has precisely dissected out the barriers to FSP attendance and then subsequently iterated upon operational methods to increase attendance using an evidence-based approach. More widely in the rabies agenda, we hope that this program of work will help facilitate international elimination efforts, particularly in SSA, where the dog ownership structure is very similar to our study site in Blantyre (14, 18, 32). Further work is required to model the financial implications of this, which will show the incremental cost-effectiveness of the new approach. This would facilitate governments to vaccinate more dogs and allow us to accelerate toward a rabies-free world.

Moreover, dog-focused public health strategies are not limited to rabies. Other dog-related disease-control efforts such as deworming dogs to prevent human echinococcosis (41) and the use of dog insecticide treatment or vaccine to prevent human Chagas disease and Leishmaniasis (40, 42, 43) share similar campaign protocols and may benefit greatly by our approach. We have shown that our approach can overcome some of the barriers to attendance to primary care clinics, and hence could have significant health care cost reductions without a reduction in accessibility in other disease scenarios.

Our study had several limitations. The trial was carried out in Blantyre city, where Mission Rabies has been working since 2015. While the familiarity of people with the charity’s efforts may have increased the success of the trial, it is expected that some people will have not attended FSPs in anticipation of the D2D component, which was a feature in previous years’ campaigns (40). Additionally, the model used to identify areas of low coverage and choice of RSP zones was carried out in real time, so there was potential for improvement. For instance, the outcome variable—vaccination coverage—was collapsed to a binary outcome, resulting in loss of information. Additionally, the model with the lowest AIC was chosen as the final model, prioritizing simplicity over choosing the model with the highest predictability. This was chosen because the final model had an area under the curve (AUC) only 3% lower than the top model but was deemed much easier to implement in similar scenarios in other cities in SSA. The distance measure used in this model was distance between the center of each zone to the nearest FSP, which may have resulted in inaccurate estimates of the effect of distance. Furthermore, using an SP-only approach results in exclusion of nonaccessible dogs, i.e., those dogs that cannot be handled by owners and unowned dogs. The proportion of nonaccessible dogs in an area should be known, and corrective measures taken, to ensure homogenously high vaccination coverage. Last, we used RSPs to increase vaccination coverage in zones where FSPs alone failed to do so. It has been shown that the use of RSPs may result in heterogeneous coverage of the areas (40). This might be a result of time constraints or inability to check whether the whole area is covered. Here we propose the use of the Worldwide Veterinary Service (WVS) app (33), which enables users to assign a working zone and enables vaccination teams to record path taken and ensure the whole working zone has been covered.

Overall, we have been able to use a data-driven approach to develop a cost-effective, scalable SP-only mass vaccination methodology, which removes the necessity for costly D2D vaccination approaches. The use of this spatially optimized FSP approach, supplemented by RSPs in areas of low population density and an effective sensitization campaign, will enable the design of successful, logistically feasible urban dog vaccination campaigns and increase scalability of rabies-control efforts in SSA and other parts of the world where the proportion of owned dogs is high. It is expected that this approach will benefit other dog-focused public health strategies.

## Materials and Methods

### Ethics Statement.

The annual rabies vaccination campaigns in Blantyre city since 2015 are part of a nonresearch public health campaign carried out by NGO Mission Rabies, supported by the Department of Animal Health and Livestock Development of the Ministry of Agriculture, Irrigation and Water Development. Prior to vaccination of owned dogs, verbal informed consent was obtained from the person presenting the dog for vaccination. In the cases where an owner could not be identified, dogs were vaccinated in accordance with Government Public Health protocol. The investigation of rabies vaccination approaches in Malawi has been approved by the University of Edinburgh Veterinary Ethics Research Committee (VERC 64/15).

### Study Site.

This study was conducted in Blantyre city, the second largest city in Malawi, with a human population of 800,264 in 2018 (10). The city covers an area of 220 km^2^, which is divided into 25 administrative wards (44). In 2015, the dog population in Blantyre city was estimated to be 45,526 based on mark resight methods (14).

Annual mass dog rabies vaccination campaigns covering the whole of Blantyre city have been run by Mission Rabies since 2015, offering free-of-charge dog rabies vaccination. For the purposes of these campaigns, the city was divided in 213 working zones, and their sizes were subjectively dictated according to an area that could be covered by a vaccination team in 1 d.

Each vaccination zone was assigned a land type based on appearance in Google Satellite Maps: 1) housing category (HS) 1 (small houses, high density), 2) HS 2 (small houses, medium density), 3) HS 3 (small houses, low density), 4) HS 4 (medium houses, ordered), 5) HS 5 (large houses, medium/low density), 6) industrial/commercial, and 7) agriculture/open space (14). For the purposes of the regression analysis described later, these were regrouped in high (*1*), medium (*2* and *4*), and low (*3* and *5* to *7*) housing density areas.

### Vaccination Approaches.

The vaccination strategy used in Blantyre city since 2015 has been described in detail by Gibson et al. (14). Briefly, a mixed approach including FSPs during the weekend followed by 3 d of D2D vaccinations had been utilized between 2015 and 2017, covering the whole city in 20 d. Despite the fact that this methodology was consistently successful in obtaining a sufficiently high vaccination coverage, the staffing challenges imposed by the labor-intensive D2D component limited the suitability of combined SP/D2D approaches to be upscaled to national rabies elimination campaigns.

### FSPs and RSPs.

To address the challenges of the combined FSP/D2D campaigns, we redesigned the 2018 vaccination campaign with the ambition to achieve adequate vaccination coverage using FSPs and RSPs only. FSPs represent predefined locations such as schools and central locations within the local community. Vaccination days and locations of FSPs are announced to the community 1 to 3 d in advance. In contrast, for RSPs, the working zone is predefined, but RSP locations are decided on the day, and multiple locations are used. More precisely, teams go to an area, identify a location, and use a loud speaker to announce that the teams are present. The teams stay near that location and do not visit any houses. Once people stop bringing dogs to them, the team moves on to the next location within the predefined working zone.

### Data-Driven Adjustment of FSP Number and Location.

Based on our previous study in Blantyre city using data on people’s FSP attendance during our 2016 campaign, the mean straight-line distance between people’s households and the nearest FSP was estimated to be 0.812 km, with an upper quartile of 1.016 km (18). The same study also showed that there is a dramatic decrease in attendance to FSPs as distance to the nearest FPS increases. Based on this, the number and location of FSPs needed to be optimized in order to reduce the distance-related barrier to FSP attendance.

Data on GPS location of all dogs seen during the 2017 D2D campaign data were used to estimate the proportion of dogs located within 0.812 km from FSP locations used during the 2017 Blantyre city campaign. Visualization of these data enabled identification of gaps where FSP locations were needed in order to increase numbers of dogs being brought to FSPs.

**Fig. 3.**
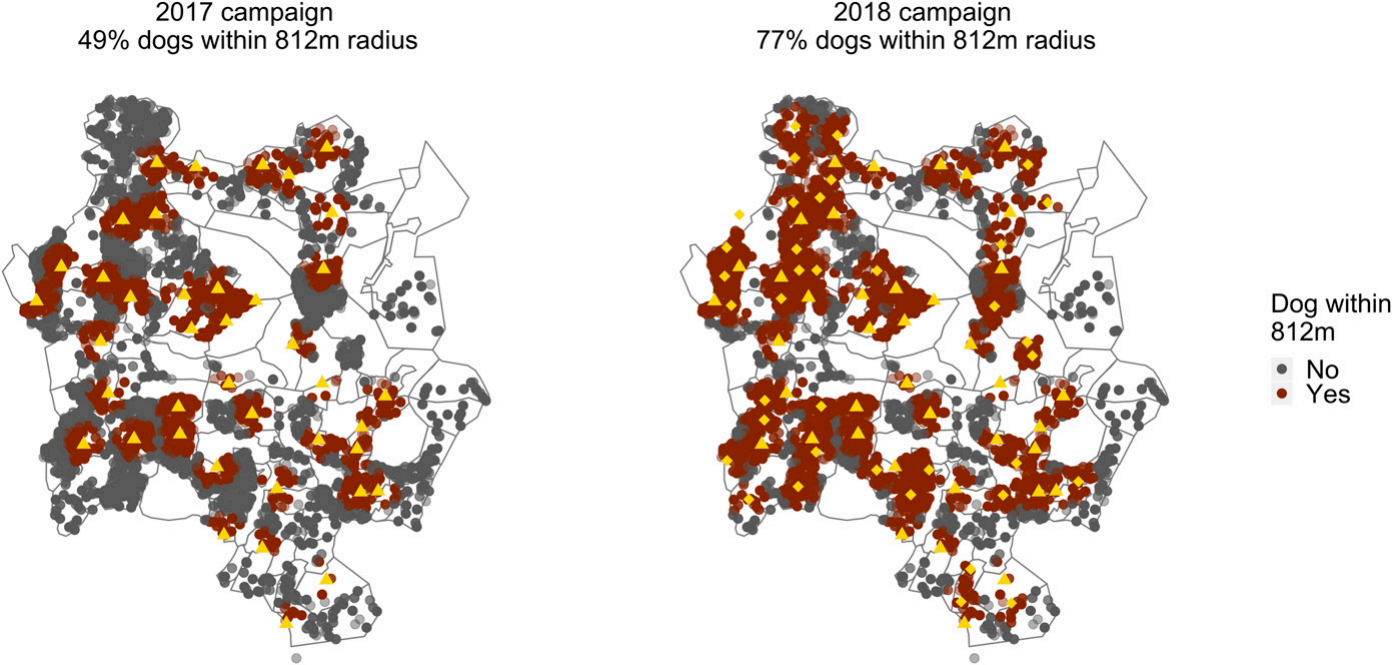
FSP locations in 2017 and 2018. FSP locations in 2017 (left, triangles) and 2018 (right, triangles and rhombi) and dogs seen during the 2017 D2D campaign colored according to whether they fall within 0.812 km of an FSP.

Original FSPs used were based upon the government rabies vaccination clinics, as these locations were well known and established. For the 2018 campaign, an analysis of FSP location and population densities was undertaken to identify regions where additional FSP locations could be potentially located. As with all potential FSP campaigns, field managers visited the site before the vaccination clinic to explain the purpose of the FSP and to obtain any appropriate permissions from the relevant authorities. Our experience from previous campaigns was that many of the dogs were brought to FSPs by children. For this reason, and because location of schools was known among the community, most of the FSPs chosen for the 2018 as well as previous campaigns were at government or private schools. Based on the 2017 dog GPS location data and staff availability, additional FSPs were chosen to increase the proportion of dogs within 0.812 km. This resulted in increasing the number of FSPs from 44 in 2017 to 77 in 2018 and the proportion of dogs within 0.812 km of an FSP from 49 to 77%. Fig. 3 shows the distribution of 2017 and 2018 FSPs and dogs seen during the 2017 D2D vaccination campaign. The 77 FSPs were divided over 4 weekends (8 vaccination days), as with previous vaccination campaigns, but no D2D vaccinations were undertaken in 2018

### Engagement with Local Community.

Mission Rabies established its annual canine rabies vaccination program in Blantyre in 2015 in collaboration with the Department of Animal Health and Livestock Development. The charity became quickly known to the public through the teams’ distinctive yellow Mission Rabies t-shirts worn during vaccination, sensitization, and education campaigns. During each campaign, our education teams visit primary schools and deliver lessons about rabies to pupils, which we have shown to be effective at increasing children’s rabies knowledge (45). This, in combination with local engagement through the delivery of rabies safety messages to the public in situ at, for example, market places, health centers, or boreholes, has increased the general public’s knowledge about rabies and how to prevent it. Therefore, the Blantyre community is highly supportive of the campaign, and the majority of dog owners are keen to get their dogs vaccinated. Indeed, in our previous study in Blantyre city, less than 1% of respondents said they did not bring their dog for vaccination at an FSP because they believed it was harmful (18).

### Increase Community Awareness.

Our previous study (18) showed that dog owners often reported that they were unaware of the vaccination campaign or that they believed puppies cannot be vaccinated as reasons for not attending FSPs. Based on this, we intensified our sensitization campaigns through increased community visits and media coverage. Teams dedicated to sensitization visited areas surrounding FSPs for 3 d before the vaccinations at these FSPs took place. The teams informed community members about rabies, its prevention, and the locations of FSPs by announcing the vaccination schedule for the upcoming weekend and distributing schedules and educational leaflets. As part of the sensitization efforts, teams informed community members that the vaccination is safe for pregnant and lactating bitches and puppies. Lastly, one of the major Malawian telecommunication companies, TNM, sponsored free text messages to their customers advertising the locations of the FSPs in their area the week before the vaccinations took place. Subsequent research in the Blantyre region supports this diverse, multimedia approach to inform local communities about our work in Blantyre, since no single sensitization approach will raise the awareness of the campaign to an entire community (46).

### Evaluation of Vaccination Coverage During the Campaign.

**Fig. 4.**
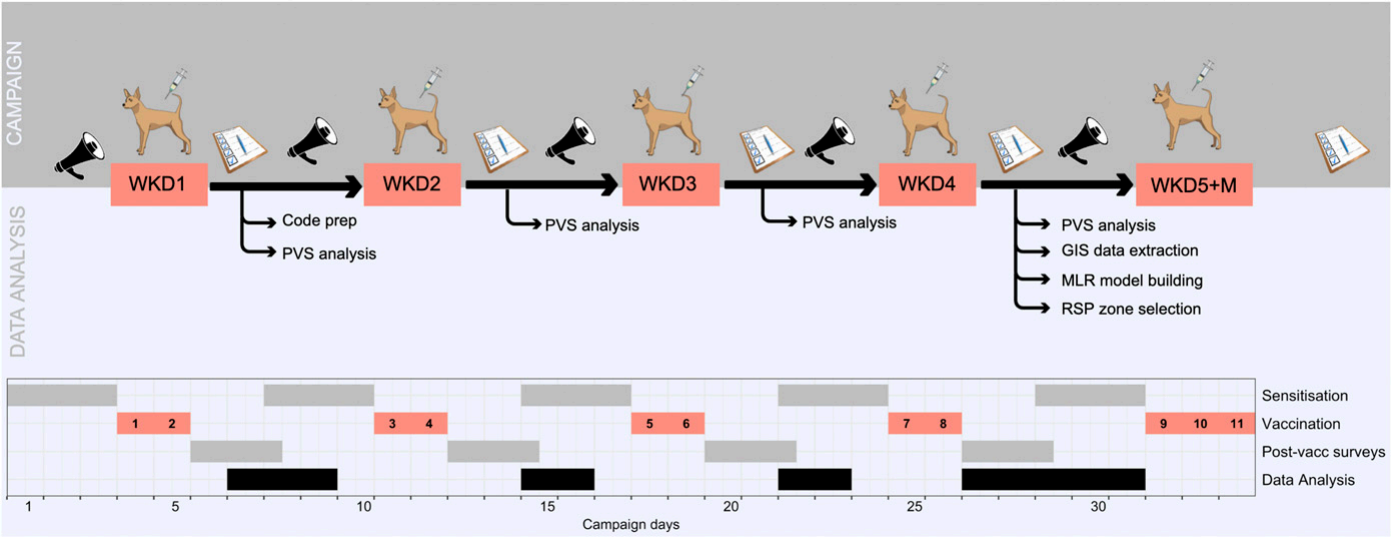
Timeline of implementation of the vaccination campaign and analysis. Timeline in days of the vaccination campaign and the data analysis that happened in parallel. GIS = Geographical Information System; M, Monday; MLR, multivariable logistic regression; WKD, weekend.

The FSP campaign took place for four consecutive weekends between April 21 and May 13, 2018. Blantyre city was split into four regions, each weekend covering FSPs in one region. At the end of each vaccination weekend, PVSs were conducted to monitor the success of the trial and assess whether adequate coverage was reached in the region covered that weekend. Each working zone was allocated a number, and zones to be surveyed were selected at random using a random number generator. A total of 25 to 30 zones were surveyed each week, resulting in 110 of 213 vaccination zones (52%) being surveyed. PVSs involved surveyors traveling through the assigned working zone, completing household questionnaires about the presence and vaccination status of dogs at every fourth house, alternating the side of the street. These surveys were carried out and analyzed in near-real time during the campaign in order to evaluate this new approach and ensure that adequate coverage (70%) would be achieved. These PVSs were carried out and analyzed during the campaign to enable us to devise a backup plan in case the use of FSPs alone was not adequate. Fig. 4 presents the timeline of implementation of the vaccination campaign and analysis.

### Choice of Working Zones that Required Roaming Vaccination Points.

The vaccination coverage after 8 d of FSPs was 65%, i.e., marginally lower than the required 70% minimum. In order to avoid returning to D2D to improve the vaccination coverage to acceptable levels, we chose to use RSPs. To optimize our RSP efficiency, we decided to target working zones with low vaccination coverage. To achieve this, we needed to identify which vaccination zones in our field site were likely to have low coverage.

The PVS data collected at the end of each vaccination weekend throughout the campaign provided data on vaccination coverage for each sampled working zone, but we had no information on vaccination coverage for the other half of the vaccination zones since they were not surveyed. To aid in classifying the rest of the vaccination zones as low or high vaccination coverage zones, we developed a multivariable logistic regression model. The model used PVS data results, i.e., coverage higher or lower than 50%, as the outcome variable. Relevant freely available Geographical Information System data, as described below, were extracted for all working zones. These were used as predictor variables. For each working zone, explanatory variables included housing density, poverty, population density, and straight-line distance to the nearest FSP from the center of the polygon. The availability of predictor variables for all vaccination zones allowed us to use the model to predict the vaccination status of all zones.

A series of models were built using all variable combinations and meaningful interaction terms. Based on our previous work on barriers to attendance to SPs in Blantyre city (18), which highlighted the interaction between distance to an FSP and poverty, the interactions investigated included 1) distance to FSP and poverty, 2) distance and housing density, and 3) distance and population density, as these three variables are all potentially associated with levels of poverty.

Fivefold cross-validation was carried out using the vtreat (47) R package to assess the predictive ability of each model by calculating the AUC using the pROC (48) R package. The final model was selected based on a combination of low AIC and high AUC.

In order to test for residual spatial autocorrelation, Moran’s *I* coefficients and *P* values on binned distance classes between all pairs of spatial coordinates of the centroids of each vaccination zone and final model residuals were calculated and visualized as a correlogram. Moran’s *I* is bounded by −1 and 1, with 0 indicating randomness, i.e., no spatial autocorrelation. *P* values <0.05 indicate that coefficients are statistically significantly different to zero and therefore the presence of spatial autocorrelation.

The final model was then used to predict whether vaccination zones were likely to have poor coverage to enable targeted RSPs. Zones categorized as industrial/commercial were removed from this process. R code used for this analysis is provided in *SI Appendix*.

Between 17 and 21 employees were available to work for three more days. Based on two employees per RSP vaccination team, we estimated a capacity to revisit at least 44 zones. These comprised zones predicted by the model to have coverage of <50% and any zones identified through surveys to have under 60% coverage. Zones were classified according to population density. Any zones in extremely low housing density areas based on population density classification and local knowledge, where very small numbers of dogs were expected to be seen, were removed to increase vaccination efficiency. In cases where teams completed their zones before the end of the day, they were given an extra zone to cover.

### Evaluation of Vaccination Coverage after the End of the Campaign.

After the end of both FSPs and RSPs, 79 randomly chosen working zones were visited and PVSs were carried out to assess if the redesigned campaign achieved a coverage of more than 70%.

### Estimation of Dogs Vaccinated by Each Method per Person per Day.

In order to compare the efficiency of the revised vaccination campaign methodology to the previously used methodology, number of dogs vaccinated per day and per person per day by each vaccination type was estimated. Vaccination teams for both FSP and D2D comprised three people: one data collector, one vaccinator, and one animal handler. At busier FSPs, more than one vaccination team was present on the same day, according to previous experience and assessment of the area. In 2018, RSP teams comprised only two people: one vaccinator and one data collector. There was a greater number of teams in previous years (2015 to 2017), mainly to due to the greater number of volunteers available, compared to 2018. Similarly, earlier campaigns lasted for 20 d, while the 2018 campaign lasted for 11 d.

### Data Collection and Analysis.

All data were collected through the WVS data collection app (33), a purpose-made smartphone app that enables team direction, data collection, and GPS location capture. All data analysis was carried out using the R statistical program (49).

### Data Sources.

#### 2017 FSP and D2D data.

During the 2017 Blantyre city D2D component of the campaign, data for every dog sited was collected, whether the dog was vaccinated or not. This included information such as signalment and vaccination status. For the purposes of this analysis, GPS locations of all dogs were used. Data were also collected for each dog vaccinated at FSPs.

#### 2018 SP and PVS data.

Data collected during the 2018 Blantyre city vaccination campaign comprised data collected during FSPs and RSPs. Data were also collected from postvaccination household questionnaires during and after the vaccination campaign as described earlier.

#### 2019 and 2020 SP and PVS data.

Based on the successful trial in 2018, the 2019 and 2020 annual vaccination campaigns were carried out using the same methodology i.e., 8 d of FSPs augmented with 3 d of RSPs. Data were also collected from postvaccination household questionnaires after the vaccination campaign as described earlier. However, no PVSs were carried out during the FSP part of the campaign.

#### Other data sources.

Population density data were sourced from WorldPop (http://www.worldpop.org.uk/). Poverty data were sourced from two WorldPop raster datasets (http://www.worldpop.org.uk/), where 2010 and 2011 estimates of proportion of people per grid square living in poverty, as defined by $1.25/d and $2/d thresholds, respectively, are available (50). Land cover data were sourced from the MASDAP Malawi Landcover 2010 Scheme I raster dataset (http://www.masdap.mw/). Land use data were sourced from Open-StreetMap data downloaded April 10, 2017 (https://www.openstreetmap.org/).

The data that support the findings of this study are available in Datasets S1–S4.

## Supplementary Material

Supplementary File

Supplementary File

Supplementary File

Supplementary File

Supplementary File

## Data Availability

All study data are included in the article and supporting information.
