# Supplementary material for: Using data-driven approaches to improve delivery of animal health care interventions for public health
Source: Proc Natl Acad Sci U S A. 2021 Jan 18;118(5):e2003722118. doi: 10.1073/pnas.2003722118 (PMC7865124; doi:10.1073/pnas.2003722118)
Supplement: Supplementary File [file pnas.2003722118.sapp.pdf]

PNAS
www.pnas.org

Supplementary Information for

Using data-driven approaches to improve delivery of animal health care interventions for public health.

Stella Mazeri, Jordana L. Burdon Bailey, Dagmar Mayer, Patrick Chikungwa, Julius Chulu, Paul Orion Grossman, Fred Lohr, Andrew D Gibson, Ian G. Handel, Barend M.deC Bronsvoort, Luke Gamble, and Richard J. Mellanby

Stella Mazeri Email: stella.mazeri@roslin.ed.ac.uk

This PDF file includes:

R code
Figures S1 to S5
Tables S1 to S4

Other supplementary materials for this manuscript include the following:

Data availability.pdf

Datasets:

1. Data for vaccinations of the 2017 and 2018 campaigns are found in "Dataset S1"

2. Data for 2018 interim post vaccination surveys are found in "Dataset S2"
3. Data for 2018 final post vaccination surveys are found in "Dataset S3"
4. Data used for the multivariable logistic regression model predicting vaccination zones with low vaccination coverage are found in "Dataset S4"

This document provides R code used for multivariable logistic regression predicting low coverage used to identify which vaccination zones to revisit using roaming static points

Load packages

```
library(tidyverse)
library(knitr)
library(kableExtra)
library(forcats)
library(rmarkdown)
library(vtreat)
library(pROC)
library(ROCR)
library(sf)
library(pgirmess)
library(bookdown)
```

Import data

```
y <- read_csv("Dataset S4.csv")
y <- y %>% mutate_at(vars(contains("4")), as.factor)
y$housedens <-
  factor(y$housedens, levels = c("Low", "Medium", "High", "Missing"))
y_model <- subset(y,!is.na(overall_coverage))
```

Five-fold cross validation set up

```
# Create the splitting plan for 5-fold cross validation
set.seed(34245)  # set seed for reproducibility
splitPlan <- kWayCrossValidation(nrow(y_model), 5, NULL, NULL)

kfold_logistic <- function(df, model) {
  df$pred_add <- NA
  for (i in 1:5) {
    split <- splitPlan[[i]]
    model_add <- glm(model, data = df, family = "binomial")
    df$pred_add[split$app] <-
      predict(model_add, newdata = df[split$app,],
              type = "response")
  }
  g <- roc(poor_coverage ~ pred_add, data = df)
  df$pred_addAUC <-
```

```
    rep(stringr::str_split_fixed(g$auc, ": ", 1), nrow(df))
  return(data.frame(
    model = as.character(model)[3],
    auc = as.numeric(df$pred_addAUC[1], 4)
  ))
}
```

Choose between numeric vs categorical versions of numerical variables

Use Area Under the Curve (AUC) and visual inspection of model outcome to choose whether to use numerical or categorical version of each numerical variable. Here we chose the keep distance to nearest sp as numeric, while we used the categorical version of poverty and population density variables.

```
## choose numeric vs cat
## keep dist2spb, pop4, pov4
m5 <-
  glm(poor_coverage ~ pop4 , data = y_model, family = "binomial")
m5lin <-
  glm(poor_coverage ~ pop , data = y_model, family = "binomial")
sjPlot::plot_model(m5)
```

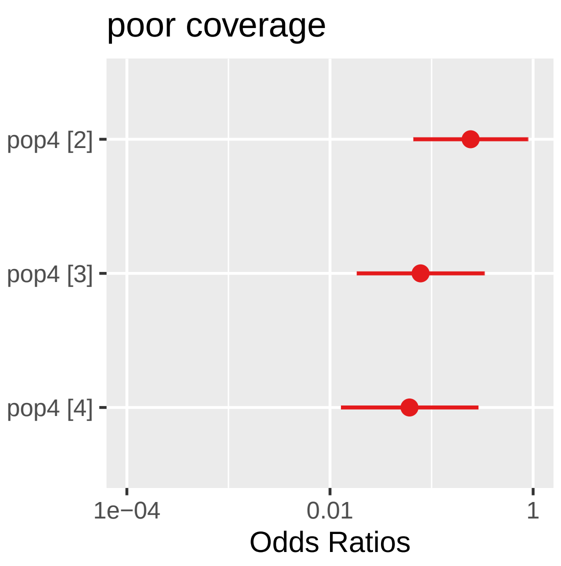

Figure S1: Population density quartiles

```
m6 <- glm(poor_coverage ~ pov4 , data = y_model, family = "binomial")
m6lin <-
  glm(poor_coverage ~ pov200 , data = y_model, family = "binomial")
sjPlot::plot_model(m6)
```

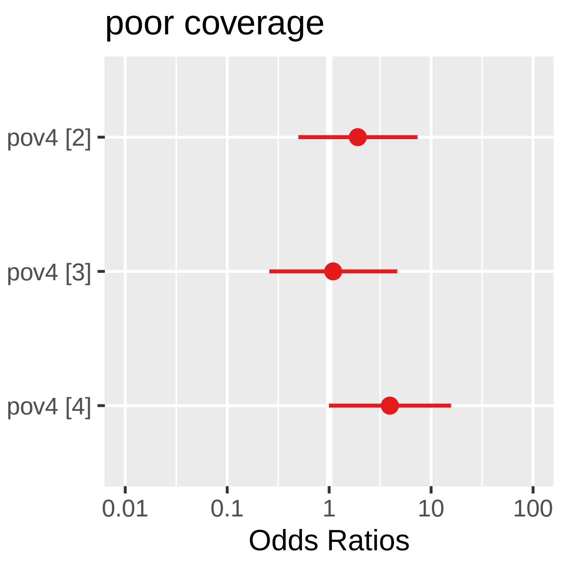

Figure S2: Poverty quartiles

```
m7 <- glm(poor_coverage ~ dist4 , data = y_model, family = "binomial")
m7lin <-
  glm(poor_coverage ~ dist2spb , data = y_model, family = "binomial")
sjPlot::plot_model(m7)
```

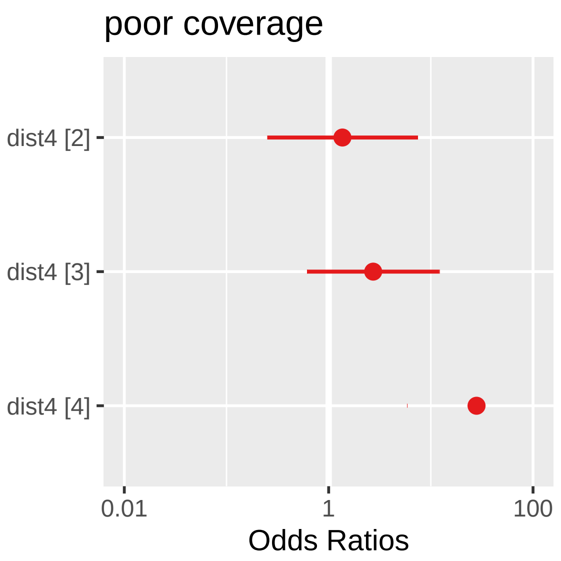

Figure S3: Distance to FSP quartiles

```
data.frame(
  rbind(
    kfold_logistic(y_model, m7$formula),
    kfold_logistic(y_model, m7lin$formula),
    kfold_logistic(y_model, m5$formula),
```

```
    kfold_logistic(y_model, m5lin$formula),
    kfold_logistic(y_model, m6$formula),
    kfold_logistic(y_model, m6lin$formula)
  )
) %>%
  kable(caption = "AUC comparison of numeric vs categorical versions of numerical variab
        booktabs = TRUE,
        escape = T,
        digits = 2)
```

Table S1: AUC comparison of numeric vs categorical versions of numerical variables

model	auc
dist4	0.79
dist2spb	0.79
pop4	0.76
pop	0.77
pov4	0.64
pov200	0.60

Choose between housing density/land type variables

Variable Descriptio has highest auc with housedens following. Variable Descriptio was considered less objective, as it was based on visual inspection of google maps images. Variable housedens was extracted from WoldPop raster data therefore considered more objective and more readily estimated for other parts of Malawi or Sub-Saharan Africa, hence chosen to be considered in the final model.

```
# choose between housing density/type variables
m2 <- glm(poor_coverage ~ fclass, data = y_model, family = "binomial")
m3 <-
  glm(poor_coverage ~ Descriptio, data = y_model, family = "binomial")
m3b <-
  glm(poor_coverage ~ housedens, data = y_model, family = "binomial")
m4 <- glm(poor_coverage ~ lc1, data = y_model, family = "binomial")

data.frame(rbind(
  kfold_logistic(y_model, m2$formula),
  kfold_logistic(y_model, m3$formula),
  kfold_logistic(y_model, m3b$formula),
  kfold_logistic(y_model, m4$formula)
)) %>%
  arrange(-auc) %>%
  kable(caption = "AUC comparison of housing density/type variables",
        booktabs = TRUE,
```

```
        escape = T,
        digits = 2)
```

Table S2: AUC comparison of housing density/type variables

model	auc
Descriptio	0.78
housedens	0.75
lc1	0.73
fclass	0.65

Variable selection for final model

The final model was selected based on lowest AIC and high auc.

```
# variables to consider: housedens, pop4, pov4, dist4
m1 <-
  glm(poor_coverage ~ housedens + pov4 + pop4 + dist2spb ,
      data = y_model,
      family = "binomial")
m1b <-
  glm(poor_coverage ~ housedens + pov4 + dist2spb ,
      data = y_model,
      family = "binomial")
m1c <-
  glm(poor_coverage ~ housedens + pop4 + dist2spb ,
      data = y_model,
      family = "binomial")
m1d <-
  glm(poor_coverage ~ pov4 + pop4 + dist2spb ,
      data = y_model,
      family = "binomial")
m1e <-
  glm(poor_coverage ~ pop4 + dist2spb ,
      data = y_model,
      family = "binomial")
m1f <-
  glm(poor_coverage ~ housedens + pov4 + pop4,
      data = y_model,
      family = "binomial")
m1g <-
  glm(poor_coverage ~ housedens + pov4,
      data = y_model,
      family = "binomial")
m1h <-
  glm(poor_coverage ~ housedens + pop4,
```

```r
      data = y_model,
      family = "binomial")
m1i <-
  glm(poor_coverage ~ pov4 + pop4, data = y_model, family = "binomial")
m1j <-
  glm(poor_coverage ~ housedens + dist2spb,
      data = y_model,
      family = "binomial")
m1k <-
  glm(
    poor_coverage ~ housedens + dist2spb + dist2spb:pov4,
    data = y_model,
    family = "binomial"
  )
m1ka <-
  glm(
    poor_coverage ~ housedens + dist2spb + dist2spb:pop4,
    data = y_model,
    family = "binomial"
  )
m1kb <-
  glm(
    poor_coverage ~ housedens + dist2spb + housedens:dist2spb,
    data = y_model,
    family = "binomial"
  )

# variable selection
a <- data.frame(
  rbind(
    kfold_logistic(y_model, m1$formula),
    kfold_logistic(y_model, m1b$formula),
    kfold_logistic(y_model, m1c$formula),
    kfold_logistic(y_model, m1d$formula),
    kfold_logistic(y_model, m1e$formula),
    kfold_logistic(y_model, m1f$formula),
    kfold_logistic(y_model, m1g$formula),
    kfold_logistic(y_model, m1h$formula),
    kfold_logistic(y_model, m1i$formula),
    kfold_logistic(y_model, m7lin$formula),
    kfold_logistic(y_model, m1j$formula),
    kfold_logistic(y_model, m1k$formula),
    kfold_logistic(y_model, m1ka$formula),
    kfold_logistic(y_model, m1kb$formula),
    kfold_logistic(y_model, m3b$formula),
    kfold_logistic(y_model, m5$formula),
```

```
    kfold_logistic(y_model, m6$formula)
  )
)

a$AIC <-
  AIC(m1, m1b, m1c, m1d, m1e, m1f, m1g,
      m1h, m1i, m7lin, m1j, m1k,
      m1ka, m1kb, m3b, m5, m6)$AIC

a <- a %>%
  mutate(AIC = round(AIC, 2),
         auc = round(auc, 2)) %>%
  arrange(AIC,-auc) %>%
  mutate(index = 1:length(AIC)) %>%
  dplyr::select(model, AIC, auc)

kable(
  a,
  caption = "Final model selection process",
  digits = 2,
  booktabs = TRUE,
  escape = T
)
```

Table S3: Final model selection process

model	AIC	auc
housedens + dist2spb	94.91	0.83
dist2spb	96.79	0.79
housedens + dist2spb + housedens:dist2spb	98.02	0.82
pop4 + dist2spb	98.10	0.83
housedens + pov4 + dist2spb	99.10	0.85
housedens + pop4 + dist2spb	100.03	0.82
housedens + dist2spb + dist2spb:pop4	100.28	0.82
housedens + dist2spb + dist2spb:pov4	100.35	0.83
pov4 + pop4 + dist2spb	101.80	0.84
housedens	102.02	0.75
housedens + pov4 + pop4 + dist2spb	104.23	0.85
housedens + pov4	105.78	0.78
pop4	105.79	0.76
housedens + pop4	105.84	0.77
pov4 + pop4	109.06	0.77
housedens + pov4 + pop4	110.00	0.78
pov4	120.12	0.64

Final model results

```
res <- sjPlot::plot_model(m1j)$data
res$colour <- rep("", nrow(res))
res$colour[res$estimate < 1] <- "A"
res$colour[res$estimate >= 1] <- "B"
res$Est4plot <- round(res$estimate, 2)
res$Sign <- rep("", nrow(res))
res$Sign[res$p.value < 0.05] <- "*"
res$Est4plot <- paste0(res$Est4plot, res$Sign)

res$term <- recode(
  res$term,
  housedensMedium = "Housing Density: Medium",
  housedensHigh = "Housing Density: High",
  dist2spb = "Distance to FSP"
)

res$term <-
  factor(
    res$term,
    levels = c(
      "Housing Density: Medium",
      "Housing Density: High",
      "Distance to FSP"
    )
  )

fm_plot <- ggplot() +
  geom_pointrange(
    data = res,
    aes(
      x = term,
      y = estimate,
      ymin = conf.low,
      ymax = conf.high
    ),
    size = 0.3
  ) +
  geom_point(data = res,
             aes(x = term, y = estimate, colour = colour),
             size = 2) +
  geom_text(
    data = res,
    vjust = -0.5,
    hjust = 0.50,
```

```
    aes(label = Est4plot, x = term, y = estimate),
    size = 4,
    colour = "darkblue"
  ) +
  geom_hline(yintercept = 1, lty = 2) +
  coord_flip() +
  xlab("Variable") + ylab("Odds Ratio (95% CI)") +
  scale_color_manual(values = c("A" = "red", "B" = "blue")) +
  scale_y_continuous(
    trans = 'sqrt',
    breaks = c(0.5, as.integer(c(10, 60, 100))),
    limits = c(0, 130)
  ) +
  theme_bw(base_size = 12) +
  theme(
    plot.title = element_text(hjust = 0.5, face = "bold"),
    axis.text = element_text(face = "bold"),
    axis.title.x = element_text(face = "bold"),
    axis.title.y = element_text(face = "bold")
  ) +
  guides(colour = FALSE)
fm_plot
```

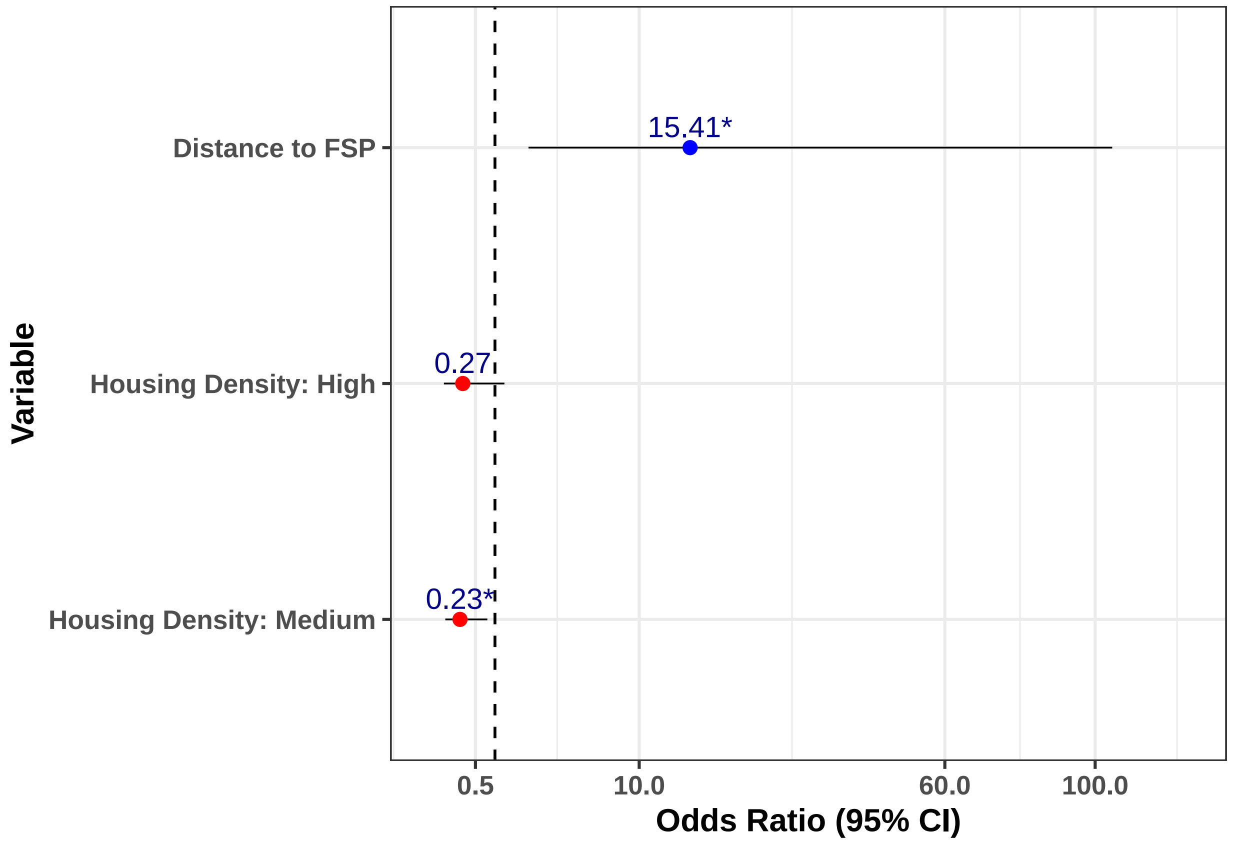

Figure S4: Regression model predicting zones with low vaccination coverage.

```
res <- res %>%
  select(term, estimate, conf.low, conf.high, std.error, p.value, p.stars) %>%
  rename("Variable" = term) %>%
  mutate(
    p.value = round(p.value, 2),
    estimate = round(estimate, 2),
    conf.low = round(conf.low, 2),
    conf.high = round(conf.high, 2),
    std.error = as.character(round(std.error, 2))
  )

res$p.value[res$p.value == 0] <- "<0.001"
res <- res %>%
  mutate(p.value = paste0(p.value, p.stars))
res <- res %>%
  mutate(`95% CI` = paste0(conf.low, "-", conf.high, 2)) %>%
  rename("Odds Ratio" = estimate,
         "SE" = std.error,
         "p-value" = p.value) %>%
  select(Variable, `Odds Ratio`, `95% CI`, SE, `p-value`)
```

```r
tibble(
  Variable = "Housing Density: Low",
  `Odds Ratio` = 1,
  `95% CI` = "Reference",
  SE = "",
  `p-value` = ""
) %>%
  bind_rows(res) %>%
  kable(
    caption = "Results of multivariable logistic regression model predicting zones
    with low vaccination coverage (CI = Confidence Interval, SE = Standard Error)",
    digits = 2,
    booktabs = TRUE,
    escape = T
  )
```

Table S4: Results of multivariable logistic regression model predicting zones with low vaccination coverage (CI = Confidence Interval, SE = Standard Error)

Variable	Odds Ratio	95% CI	SE	p-value
Housing Density: Low	1.00	Reference		
Housing Density: Medium	0.23	0.07-0.782	0.63	0.02*
Housing Density: High	0.27	0.05-1.32	0.81	0.1
Distance to FSP	15.41	2.26-105.182	0.98	0.01**

Calculate cut-off to predict low coverage areas

```r
prob <- predict(m1j, newdata = y_model, type = "response")
pred <- prediction(prob, y_model$poor_coverage)
perf <- performance(pred, measure = "tpr", x.measure = "fpr")
auc <- performance(pred, measure = "auc")
auc <- auc@y.values[[1]]

opt_cut_off = function(perf, pred) {
  cut.ind = mapply(
    FUN = function(x, y, p) {
      d = (x - 0) ^ 2 + (y - 1) ^ 2
      ind = which(d == min(d))
      c(cutoff = p[[ind]])
    },
    perf@x.values,
    perf@y.values,
    pred@cutoffs
```

```
  )
}

cut_off <- opt_cut_off(perf, pred)
```

Add column with vaccinations zones predicted by model to have low coverage

```
prob <- predict(m1j, newdata = y, type = "response")
y$pred <- as.numeric(prob > cut_off)
```

Test for residual spatial autocorrelation using Moran's I test

Compute Moran's coefficients on binned distance classes from spatial coordinates of the centroids of each vaccination zone and model residuals and visualise results as a correlogram. Moran's I is bounded by -1 and 1, with 0 indicating randomness, i.e. no spatial autocorrelation. P-values <0.05 indicate that coefficients statically significantly different to zero and therefore presence of spatial autocorrelation. There is no evidence of spatial autocorrelation here.

```
#Transform Longitute Latitute to
df_sf = st_as_sf(y_model[, c("Longitude", "Latitude")],
                 coords = c("Longitude", "Latitude"),
                 crs = "+proj=longlat +datum=WGS84")
#https://epsg.io/20936-1117
df_sf <- st_transform(df_sf, crs = "+init=epsg:20936")
df_sf <- st_coordinates(df_sf) %>% as.data.frame()

# Compute correlogram of the residuals
cor_r <- correlog(
  coords = df_sf,
  z = m1j$residuals,
  method = "Moran",
  nbclass = 10
)


correlogram <- as.data.frame(cor_r)

# Plot correlogram
a <- ggplot(correlogram, aes(x = dist.class, y = coef)) +
  geom_hline(yintercept = 0, col = "grey") +
  geom_line(col = "steelblue") +
  geom_text(aes(
    x = dist.class + 0.003,
    y = coef + 0.009,
    label = round(p.value, 3)
```

```
  ), colour = "red") +
  geom_point(col = "steelblue") +
  ylim(c(-0.15, 0.15)) +
  labs(
    x = "Distance (m)",
    y = "Moran's coefficient",
    title = "Correlogram",
    subtitle = "P-values in red"
  ) +
  theme_classic(base_size = 16)
a
```

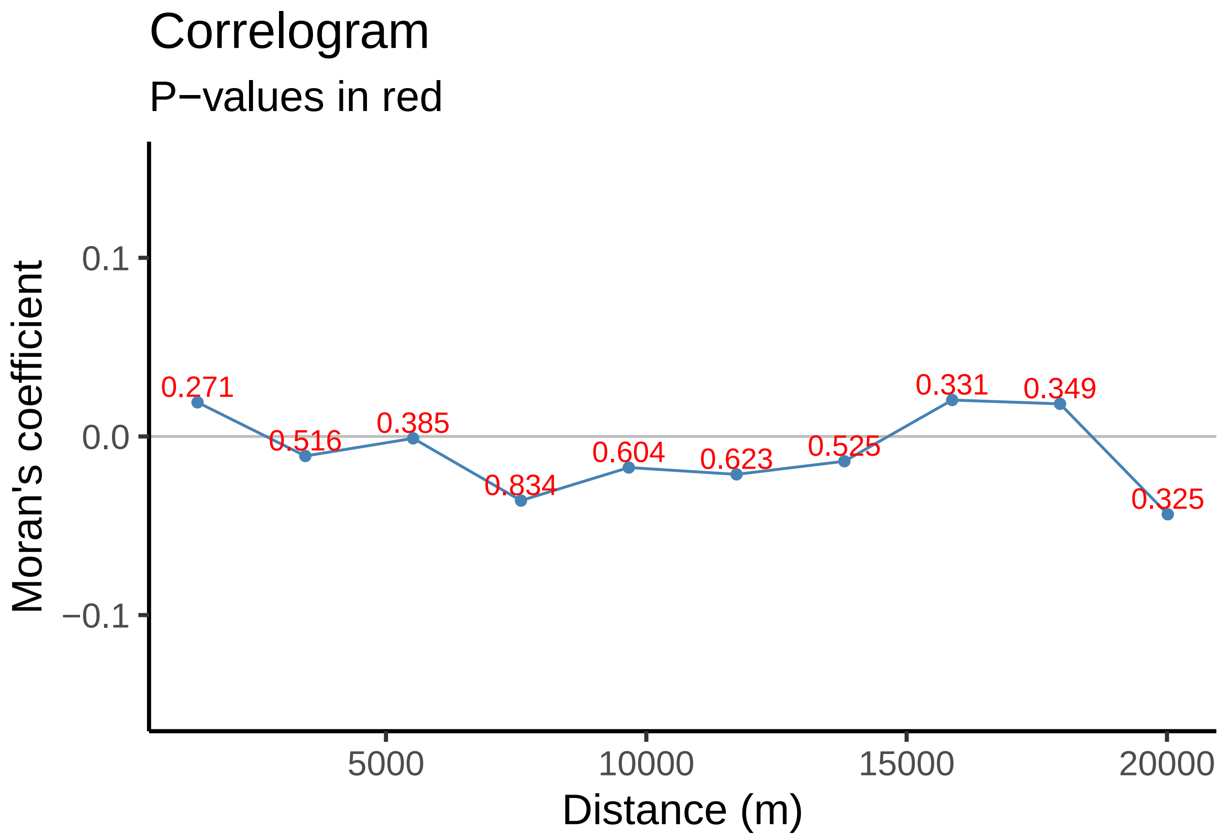

Figure S5: Correlogram of Moran's I coefficients
